# Supplementary material for: Application of an EMG-Rehabilitation Robot in Patients with Post-Coronavirus Fatigue Syndrome (COVID-19)—A Feasibility Study
Source: Int J Environ Res Public Health. 2022 Aug 20;19(16):10398. doi: 10.3390/ijerph191610398 (PMC9407702; doi:10.3390/ijerph191610398)
Supplement: Supplementary file 1 [file ijerph-19-10398-s001.zip › ijerph-1820811-supplementary.pdf]

Intervention Group (1st figure – pre – intervention, 2nd figure – post intervention)

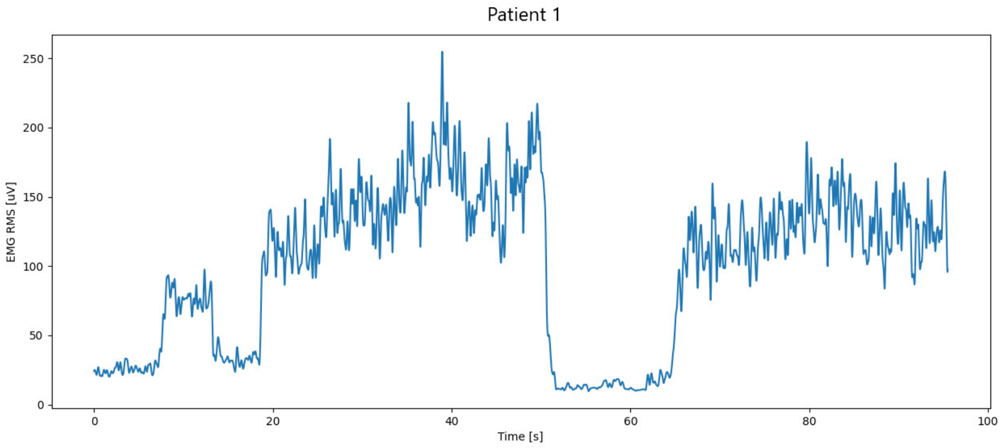

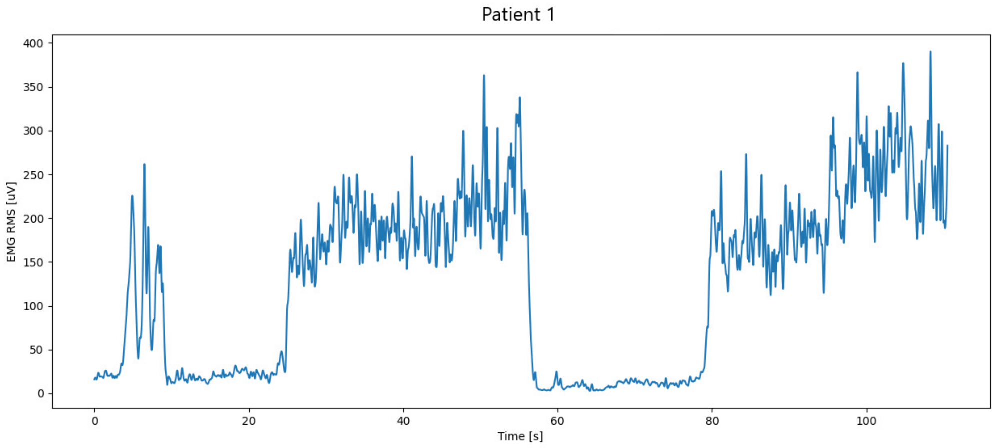

Patient 2

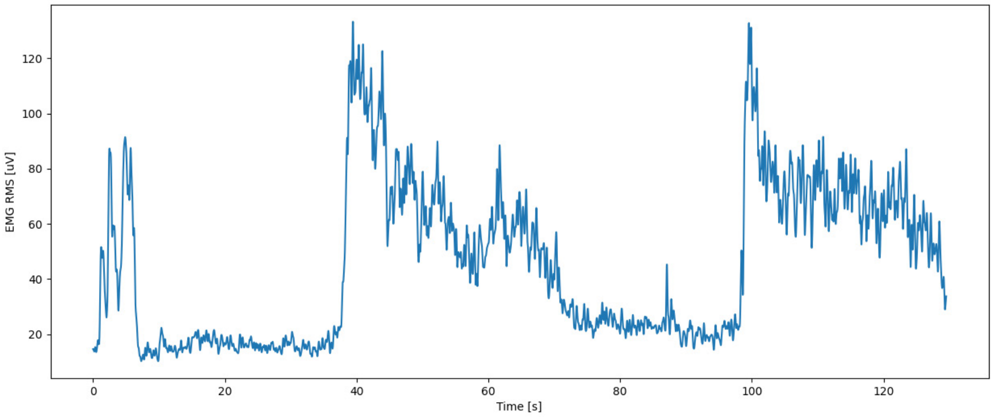

Patient 2

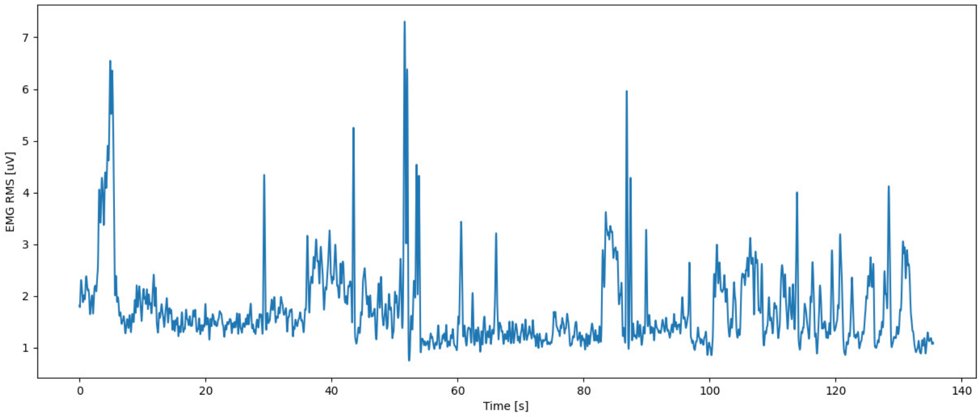

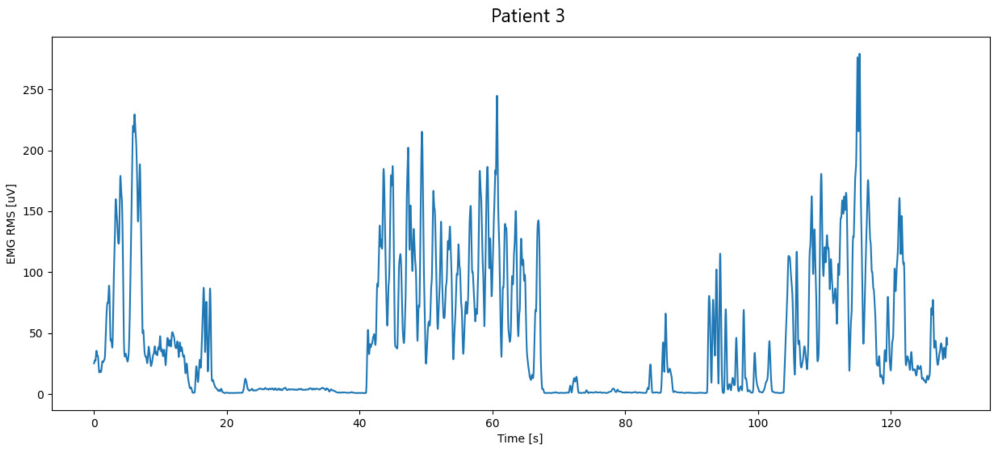
Patient 3
EMG RMS [uV]
Time [s]

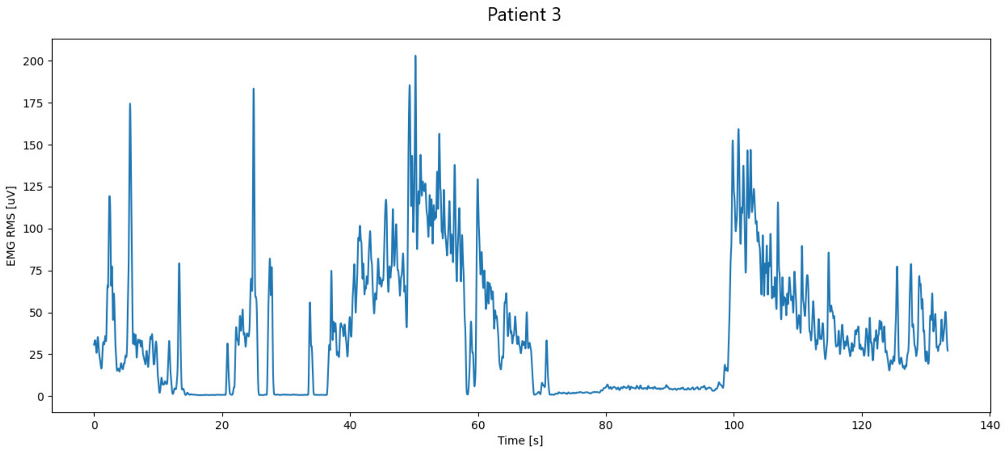
Patient 3
EMG RMS [uV]
Time [s]

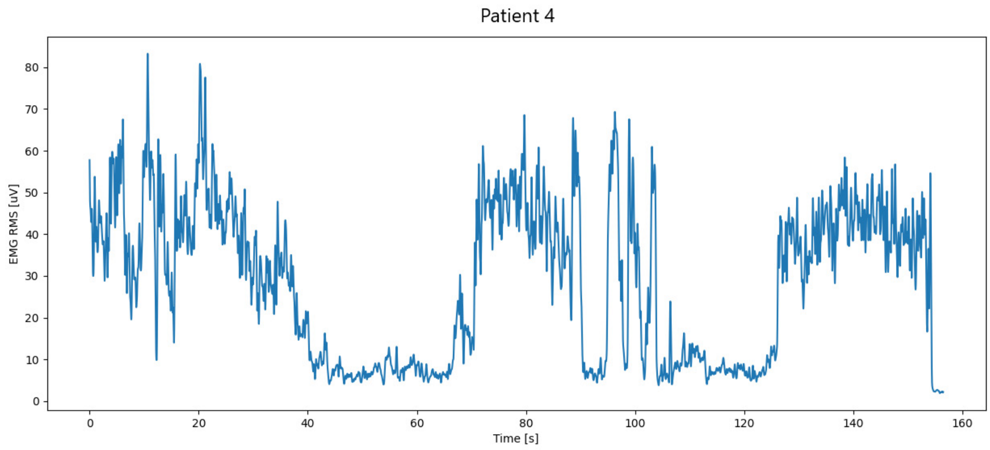
Patient 4
EMG RMS [uV]
0
10
20
30
40
50
60
70
80
0
20
40
60
80
100
120
140
160
Time [s]

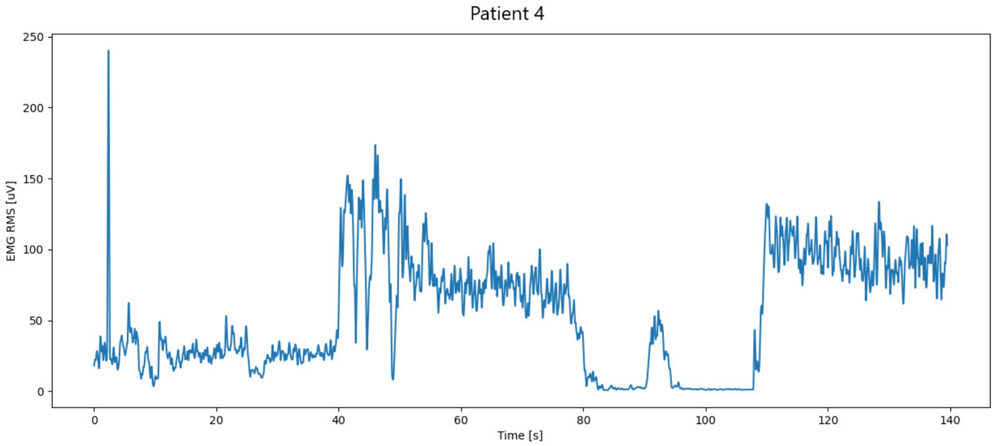
Patient 4
EMG RMS [uV]
0
50
100
150
200
250
0
20
40
60
80
100
120
140
Time [s]

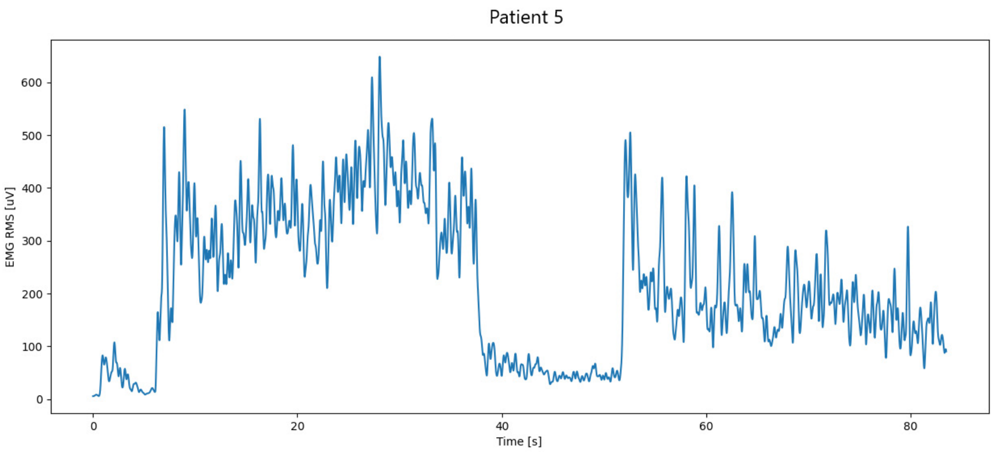
Patient 5
EMG RMS [uV]
Time [s]
0
100
200
300
400
500
600
0
20
40
60
80

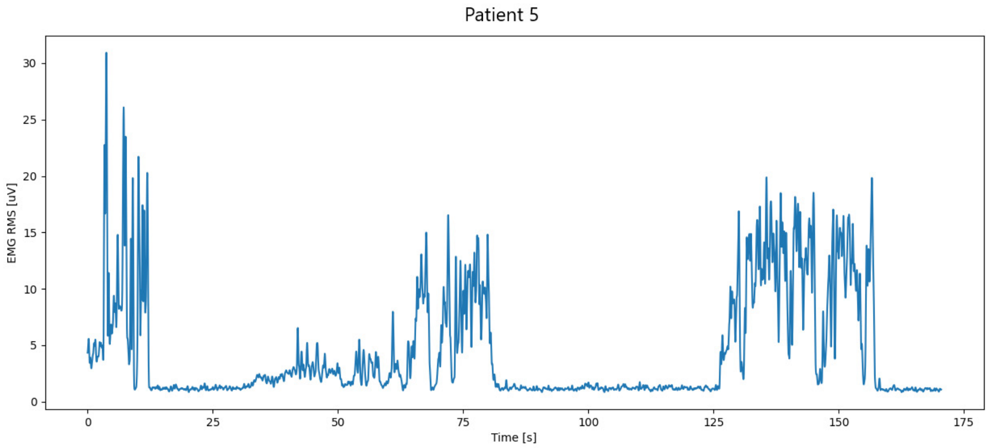
Patient 5
EMG RMS [uV]
Time [s]
0
5
10
15
20
25
30
0
25
50
75
100
125
150
175

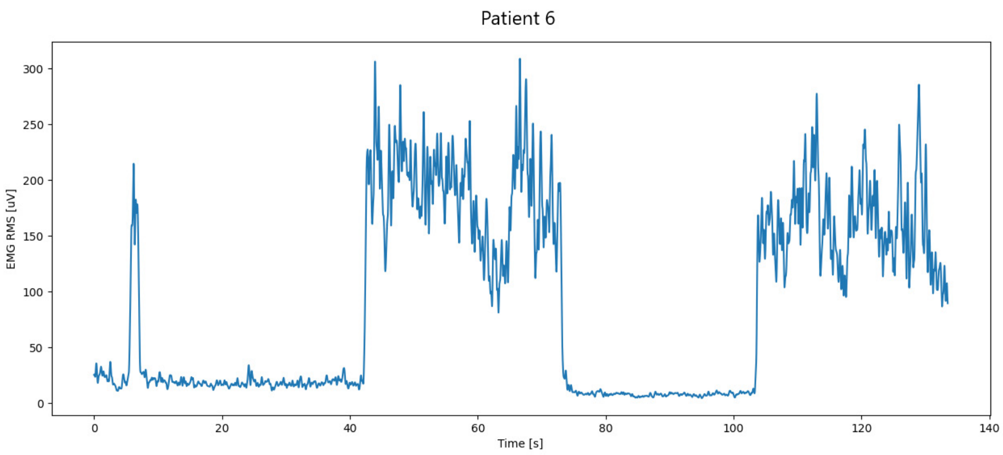

Patient 6
EMG RMS [uV]
Time [s]

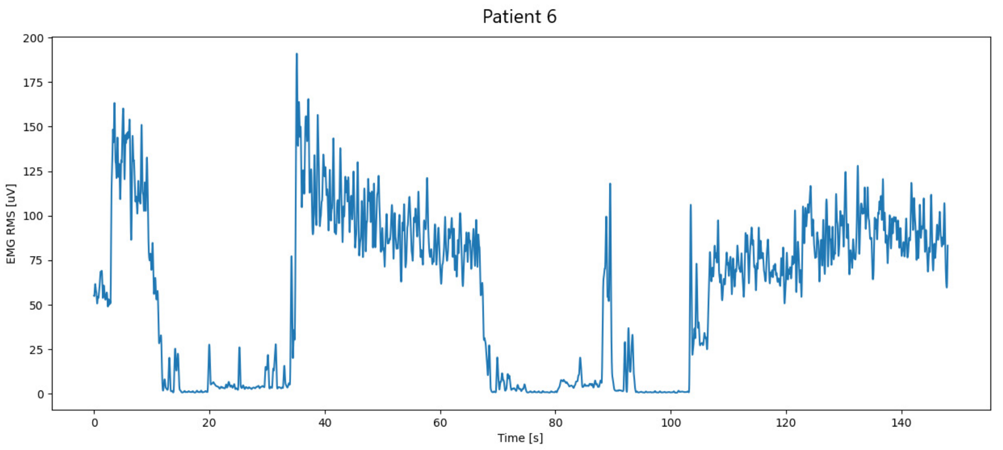

Patient 6
EMG RMS [uV]
Time [s]

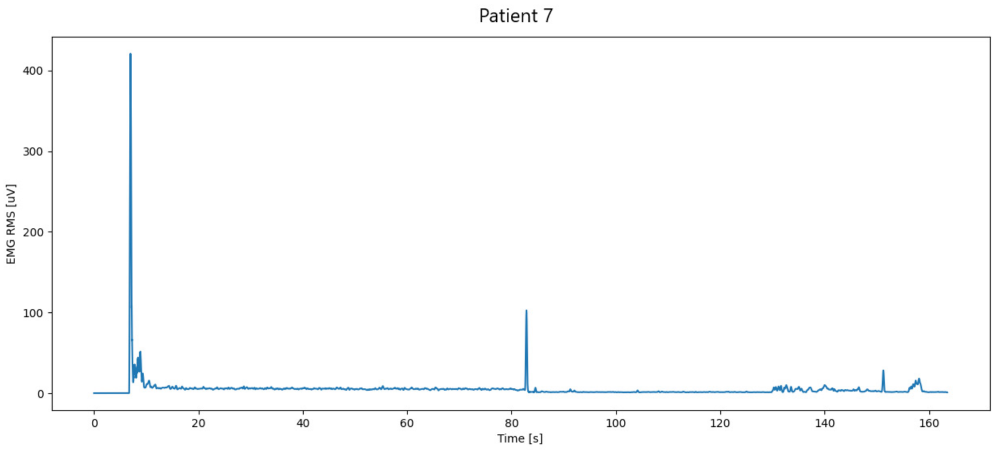
Patient 7
EMG RMS [uV]
Time [s]
0
100
200
300
400
0
20
40
60
80
100
120
140
160

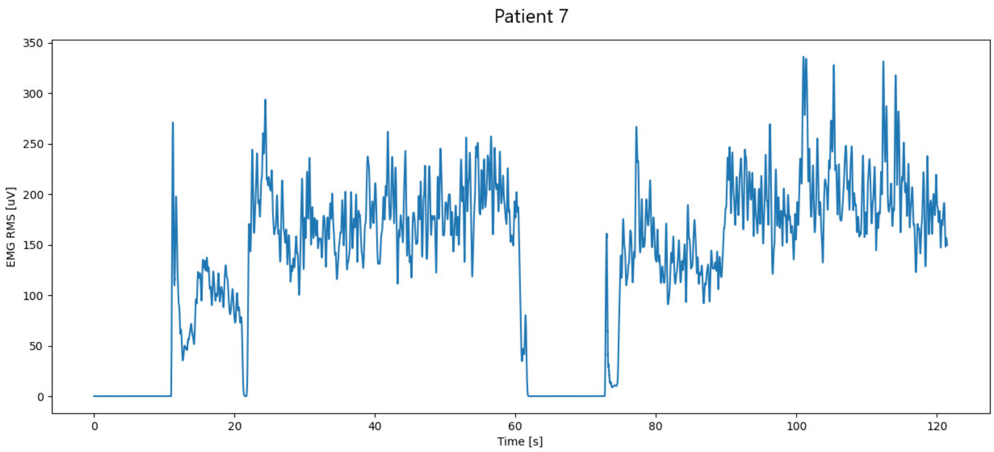
Patient 7
EMG RMS [uV]
Time [s]
0
50
100
150
200
250
300
350
0
20
40
60
80
100
120

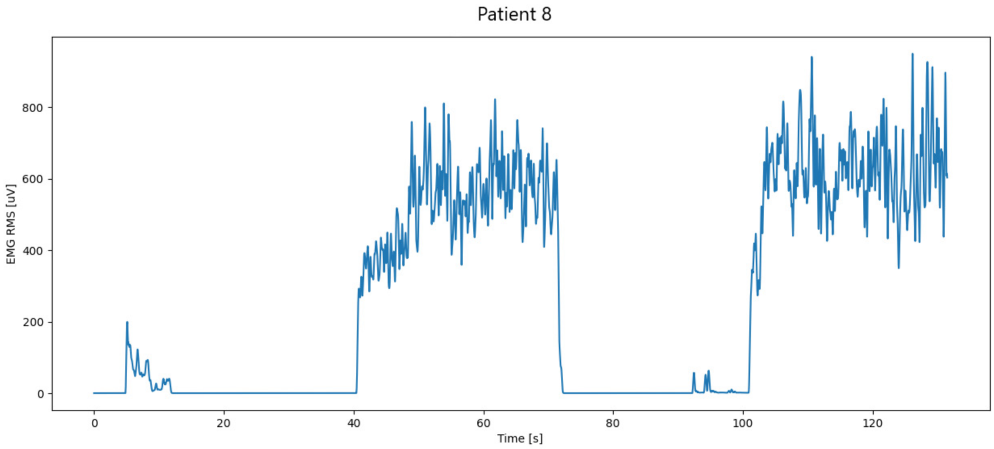
Patient 8
EMG RMS [uV]
Time [s]
0
200
400
600
800
0
20
40
60
80
100
120

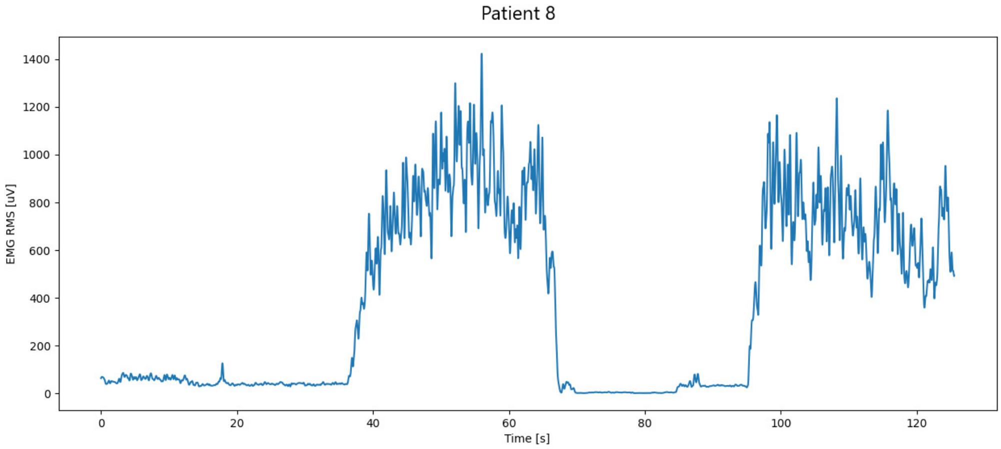
Patient 8
EMG RMS [uV]
Time [s]
0
200
400
600
800
1000
1200
1400
0
20
40
60
80
100
120

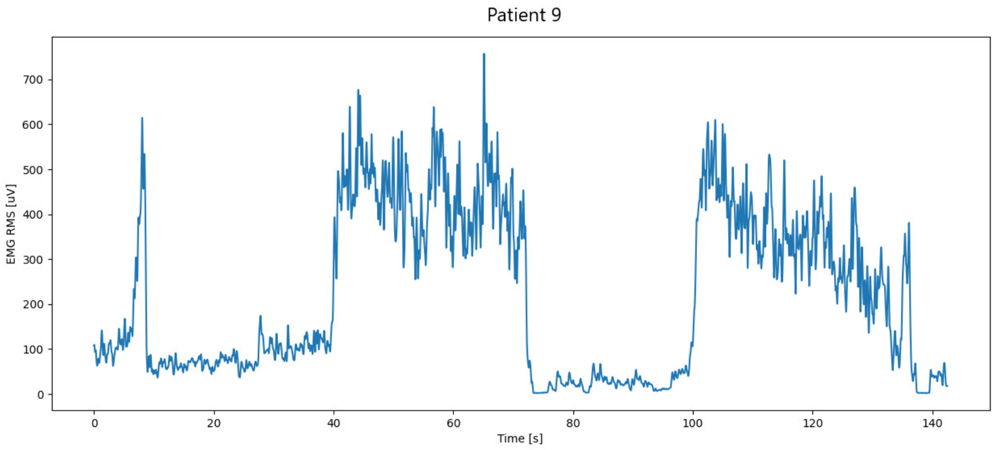

Patient 9
EMG RMS [uV]
Time [s]

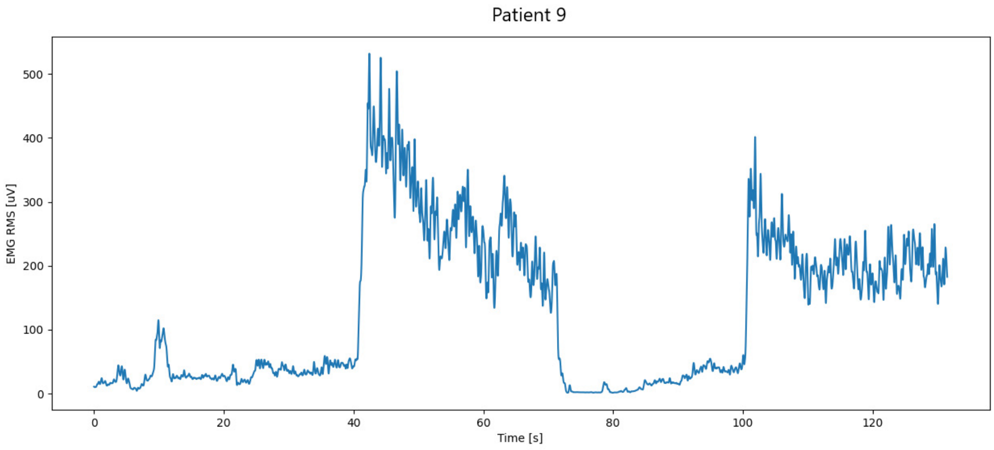

Patient 9
EMG RMS [uV]
Time [s]

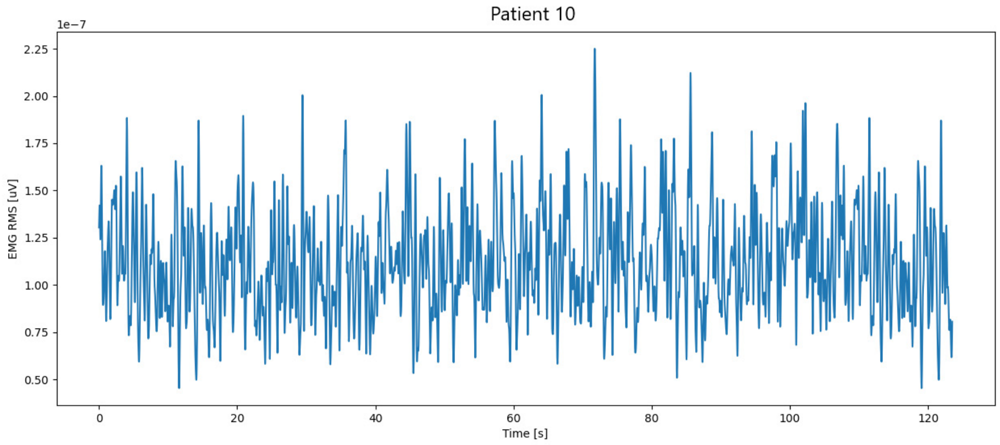
Patient 10
1e−7
2.25
2.00
1.75
1.50
1.25
1.00
0.75
0.50
EMG RMS [uV]
0
20
40
60
80
100
120
Time [s]

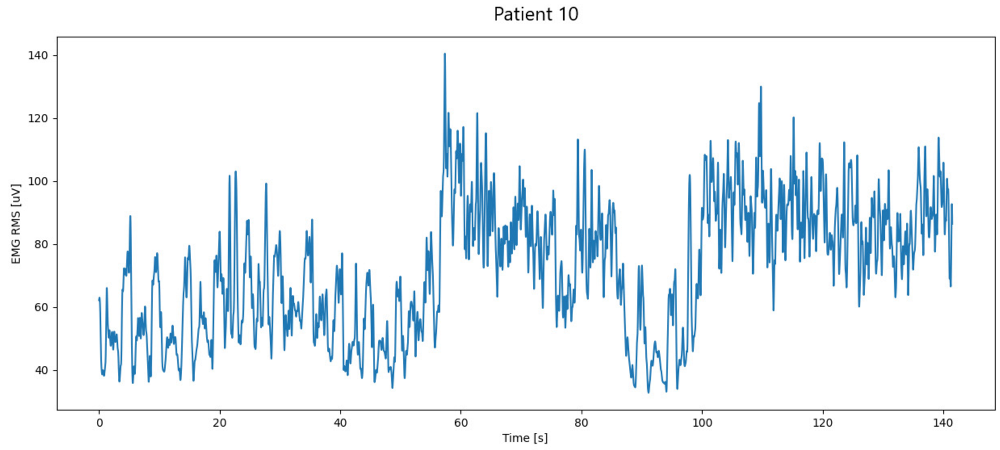
Patient 10
140
120
100
80
60
40
EMG RMS [uV]
0
20
40
60
80
100
120
140
Time [s]

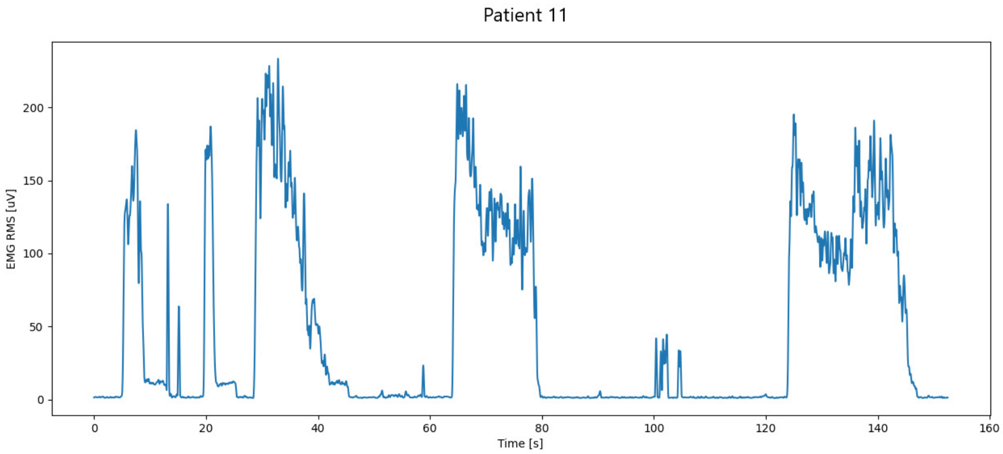
Patient 11
EMG RMS [uV]
Time [s]
0
50
100
150
200
0
20
40
60
80
100
120
140
160

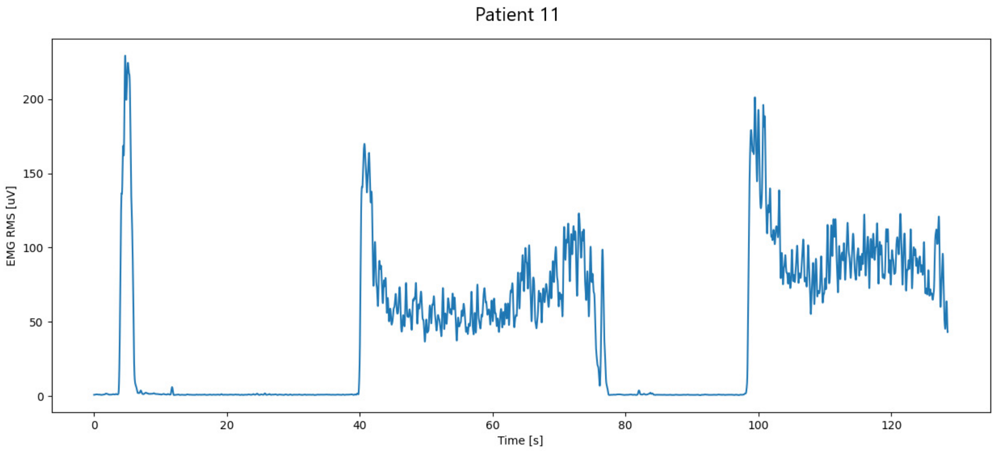
Patient 11
EMG RMS [uV]
Time [s]
0
50
100
150
200
0
20
40
60
80
100
120

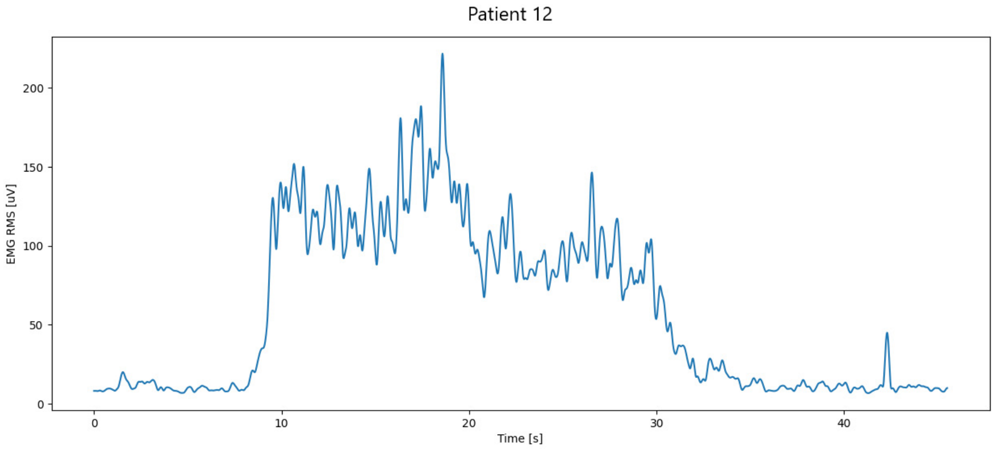
Patient 12
EMG RMS [uV]
Time [s]
0
50
100
150
200
0
10
20
30
40

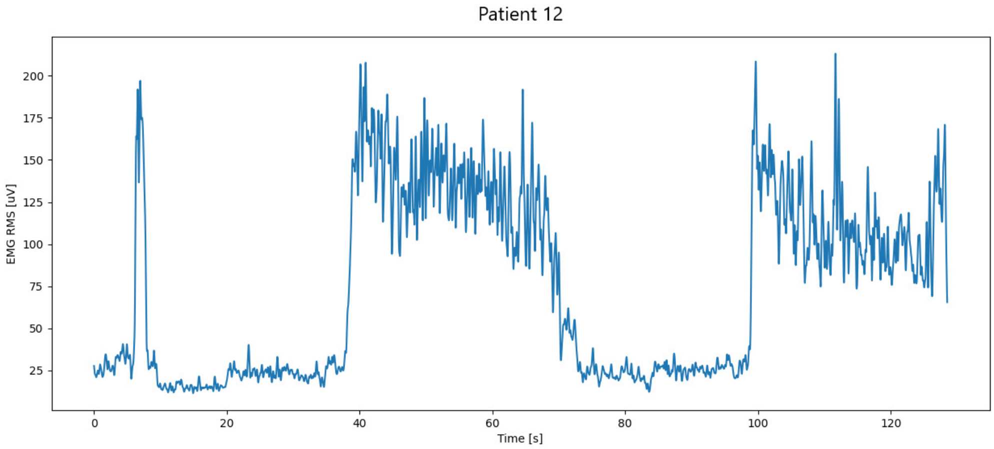
Patient 12
EMG RMS [uV]
Time [s]
25
50
75
100
125
150
175
200
0
20
40
60
80
100
120

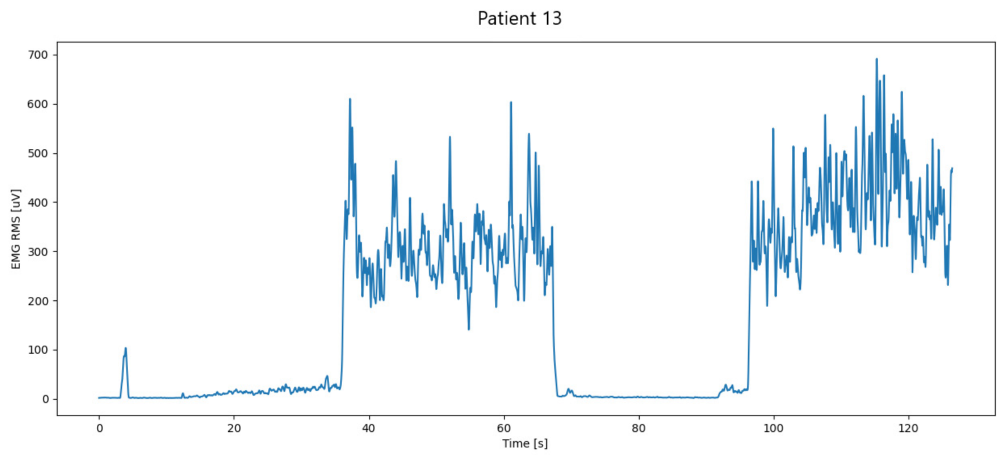
Patient 13
EMG RMS [uV]
Time [s]
0
100
200
300
400
500
600
700
0
20
40
60
80
100
120

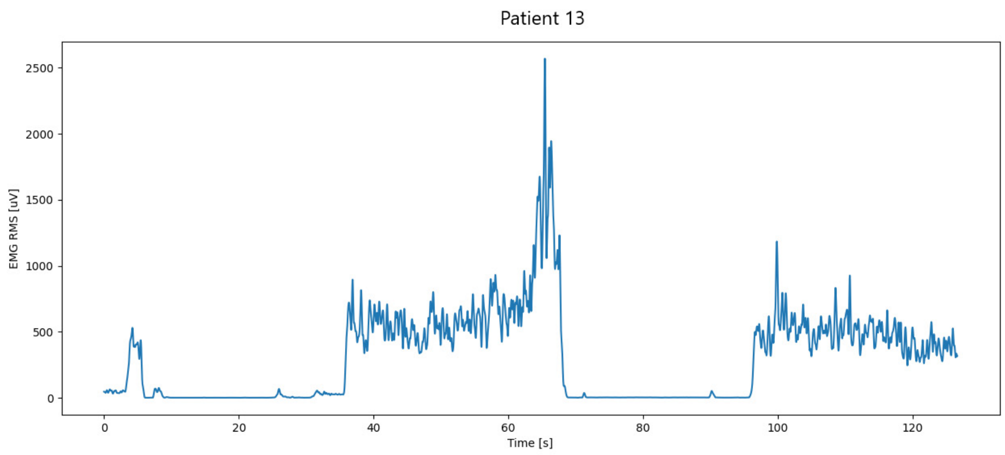
Patient 13
EMG RMS [uV]
Time [s]
0
500
1000
1500
2000
2500
0
20
40
60
80
100
120

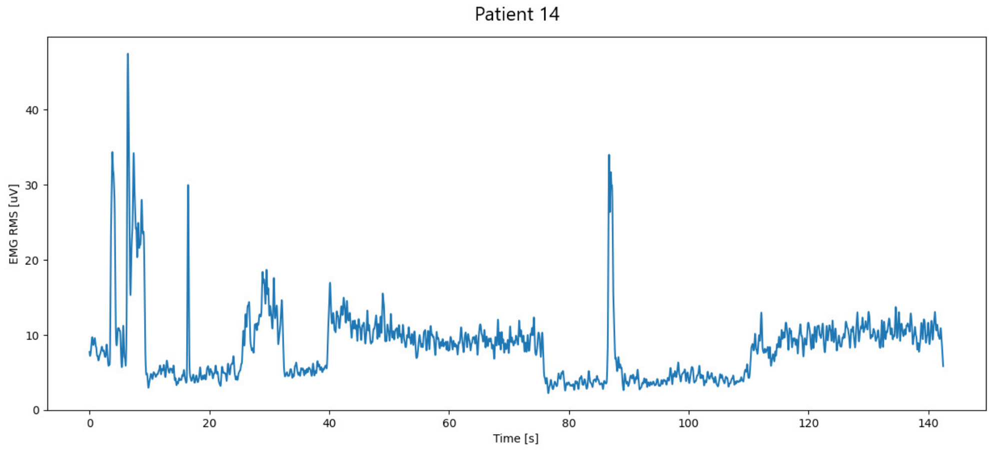

Patient 14
EMG RMS [uV]
Time [s]
0
10
20
30
40
0
20
40
60
80
100
120
140

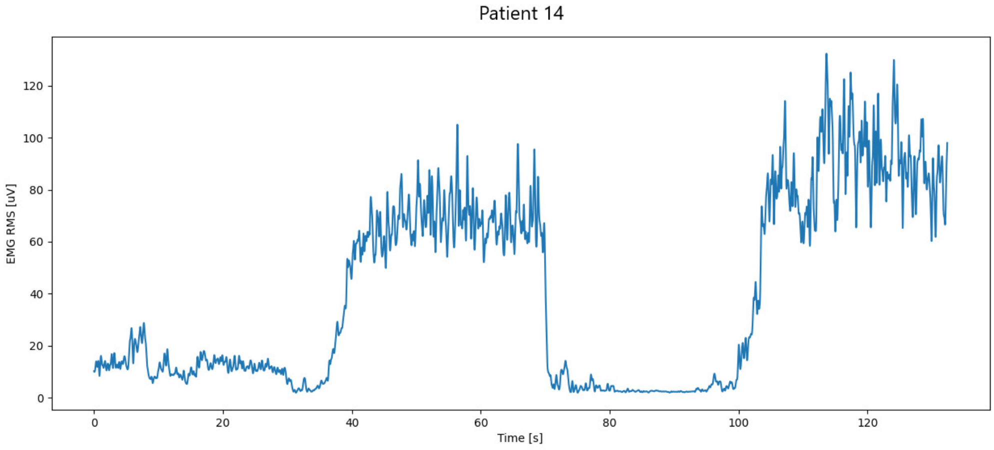

Patient 14
EMG RMS [uV]
Time [s]
0
20
40
60
80
100
120
0
20
40
60
80
100
120

Control Group (1st figure – pre – intervention, 2nd figure – post intervention)

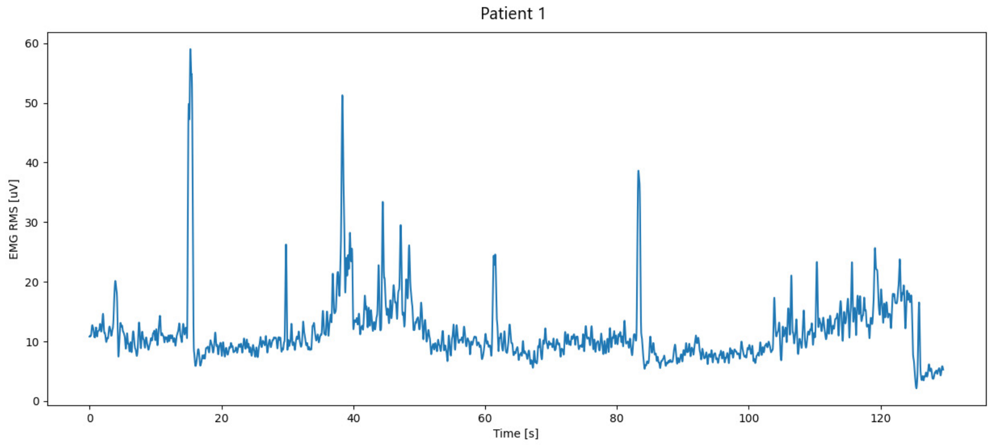

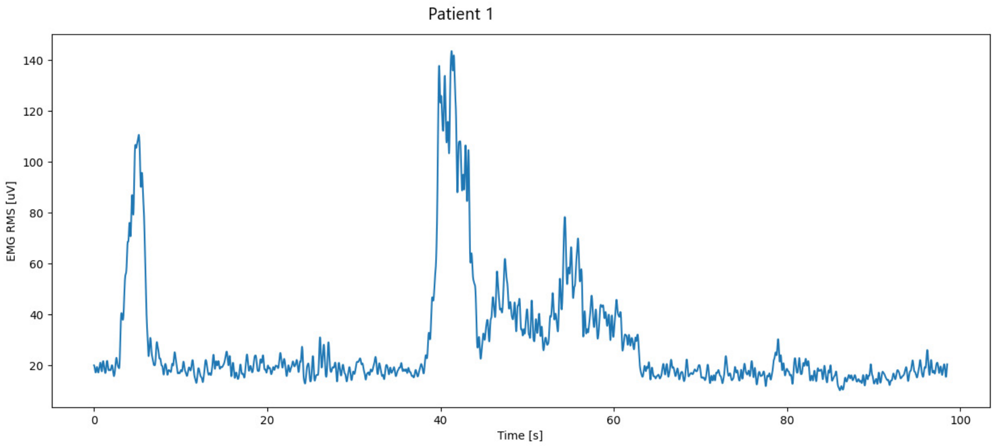

Patient 2

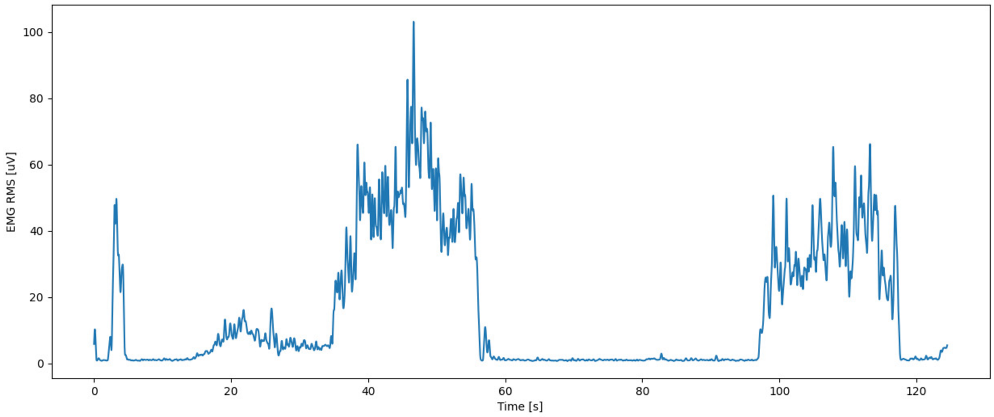

Patient 2

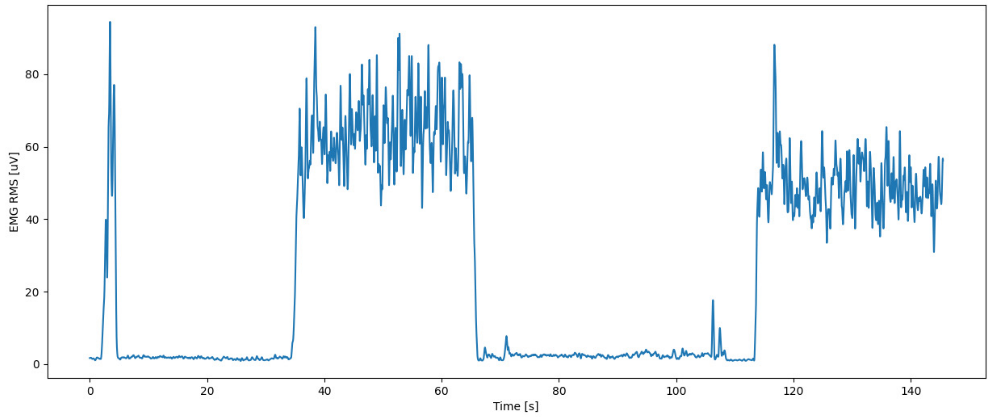

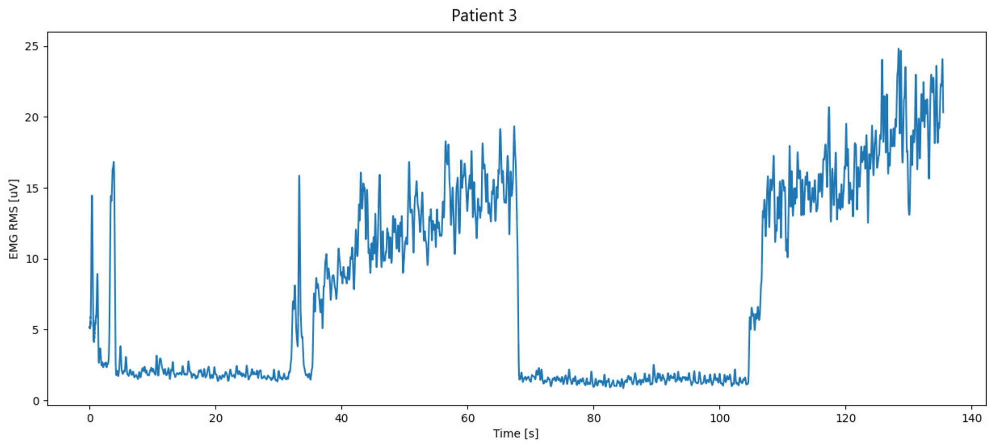

Patient 3
EMG RMS [uV]
Time [s]
0
5
10
15
20
25
0
20
40
60
80
100
120
140

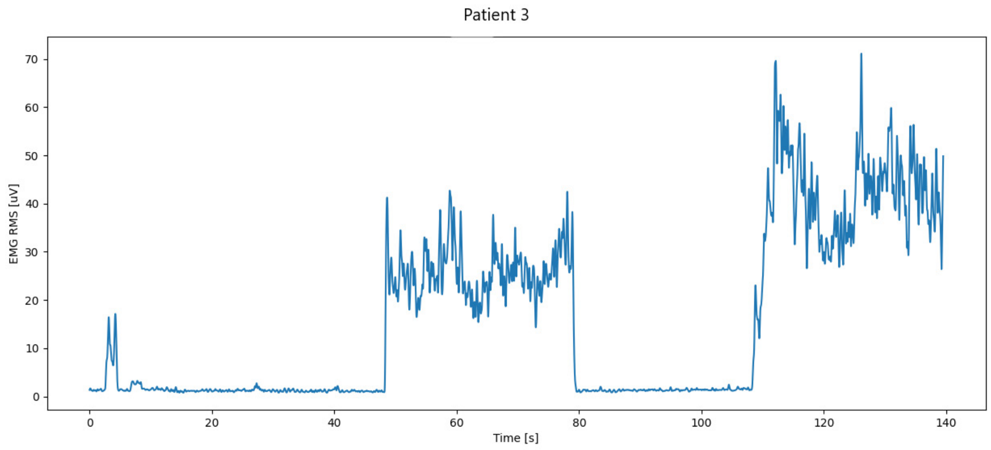

Patient 3
EMG RMS [uV]
Time [s]
0
10
20
30
40
50
60
70
0
20
40
60
80
100
120
140

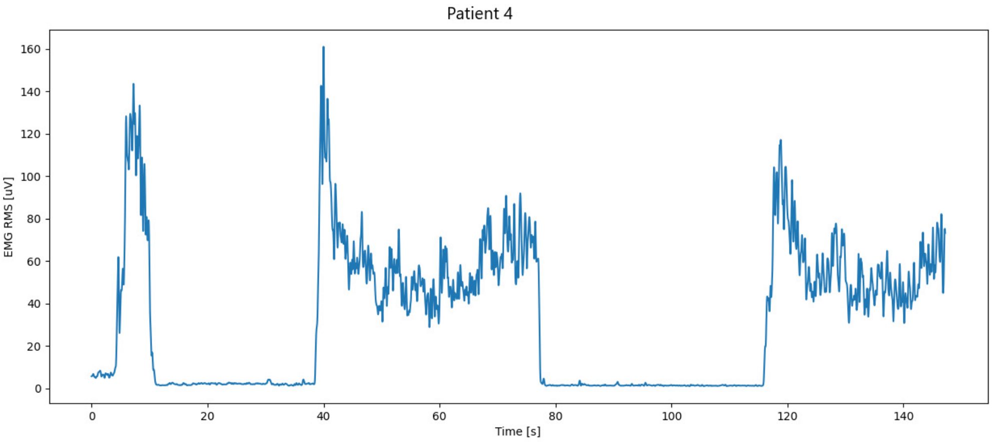
Patient 4
EMG RMS [uV]
Time [s]
0
20
40
60
80
100
120
140
160

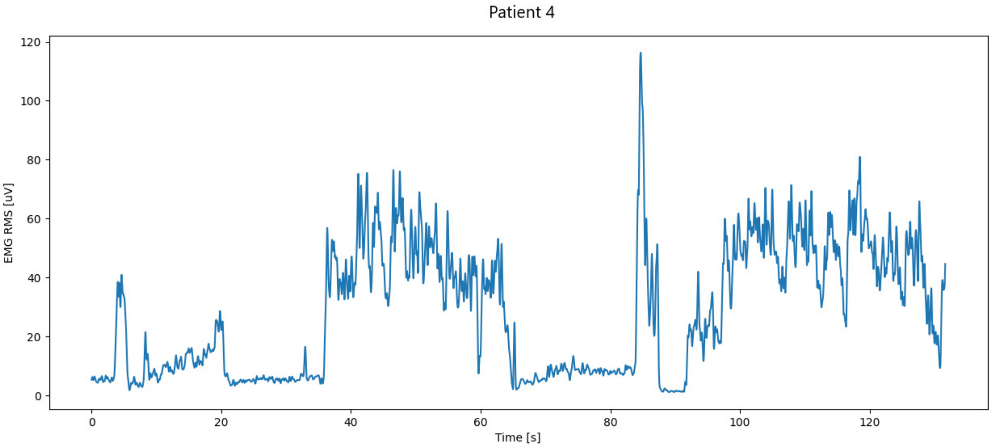
Patient 4
EMG RMS [uV]
Time [s]
0
20
40
60
80
100
120

Patient 5

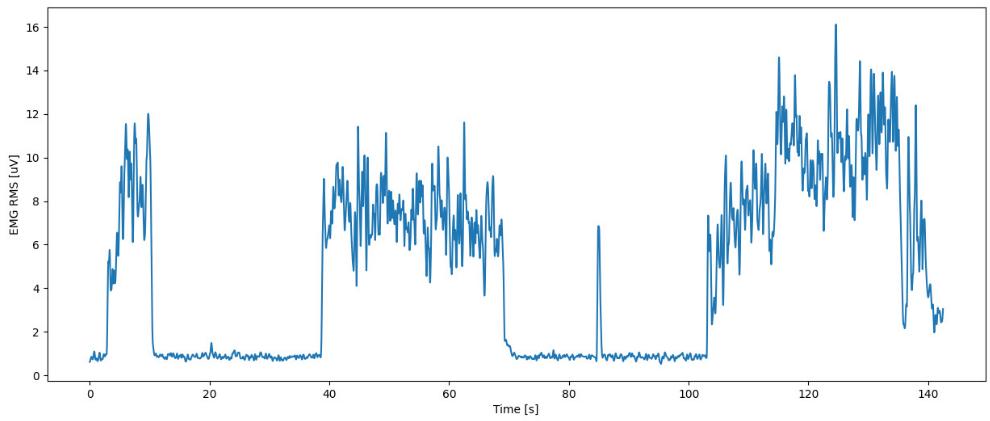

Patient 5

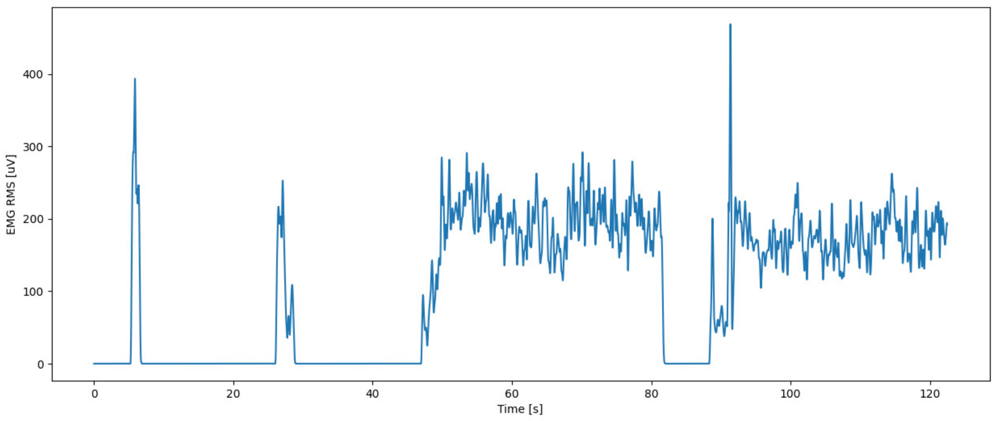

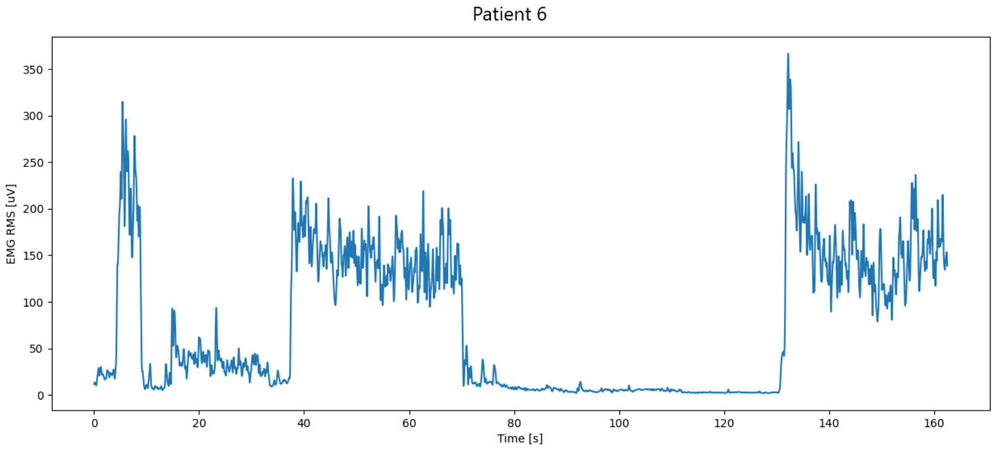

Patient 6
EMG RMS [uV]
Time [s]

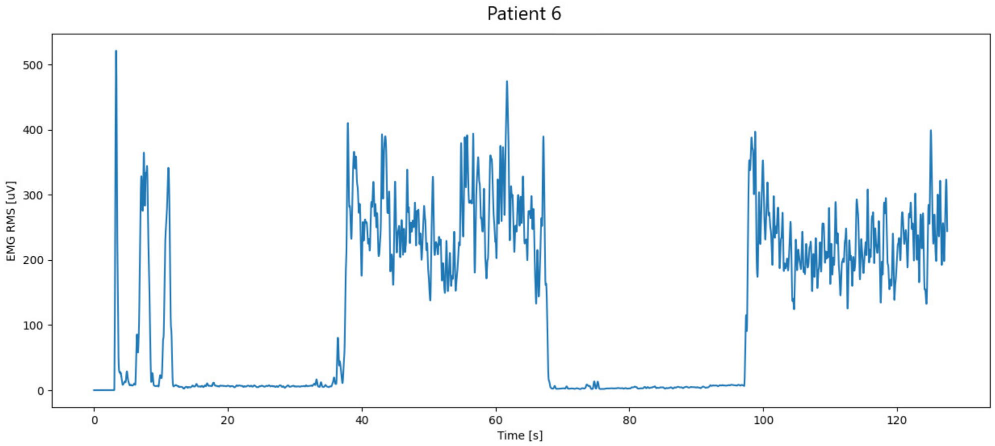

Patient 6
EMG RMS [uV]
Time [s]

Patient 7

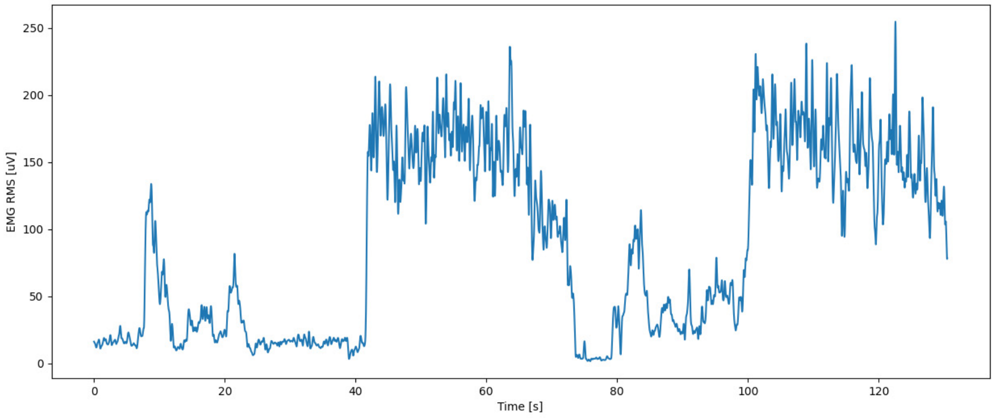

Patient 7

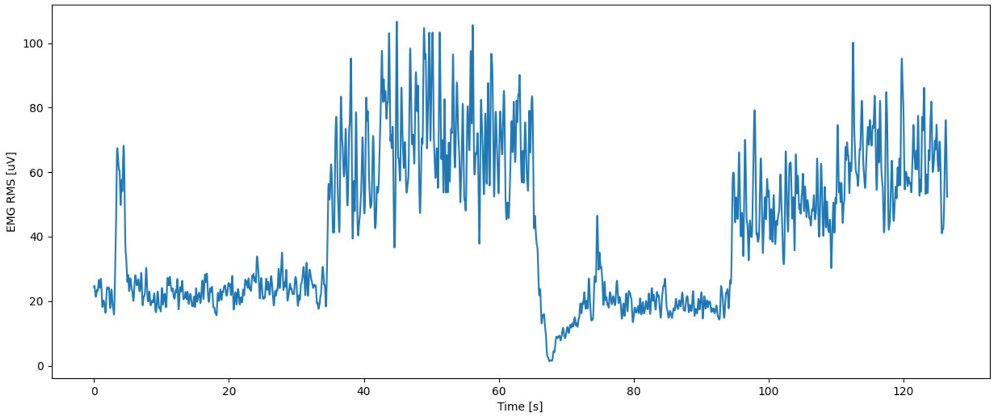

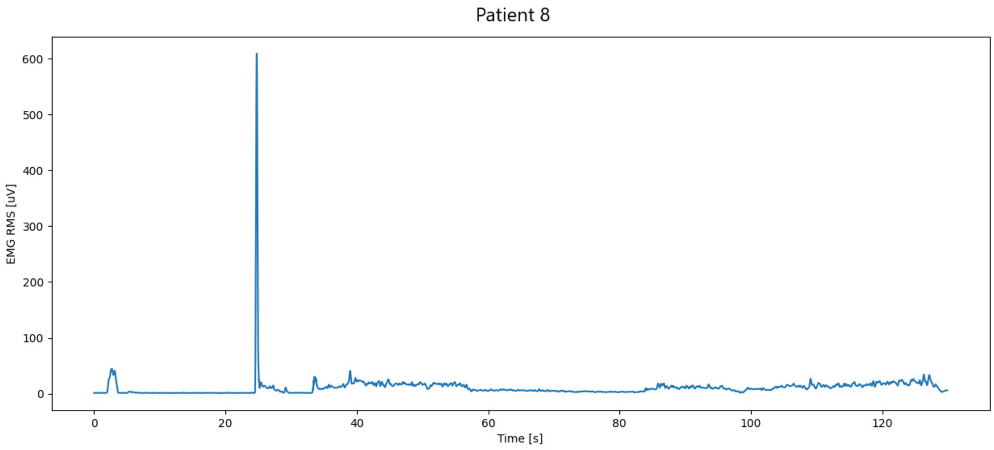

Patient 8
EMG RMS [uV]
Time [s]

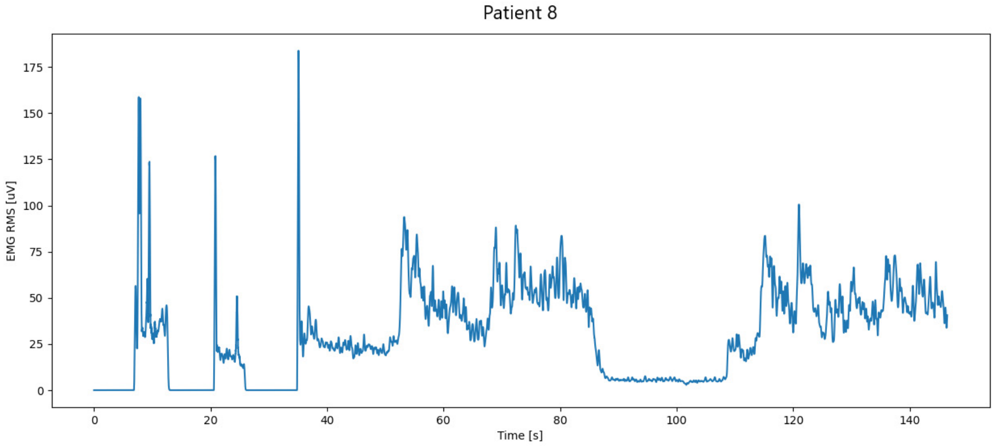

Patient 8
EMG RMS [uV]
Time [s]

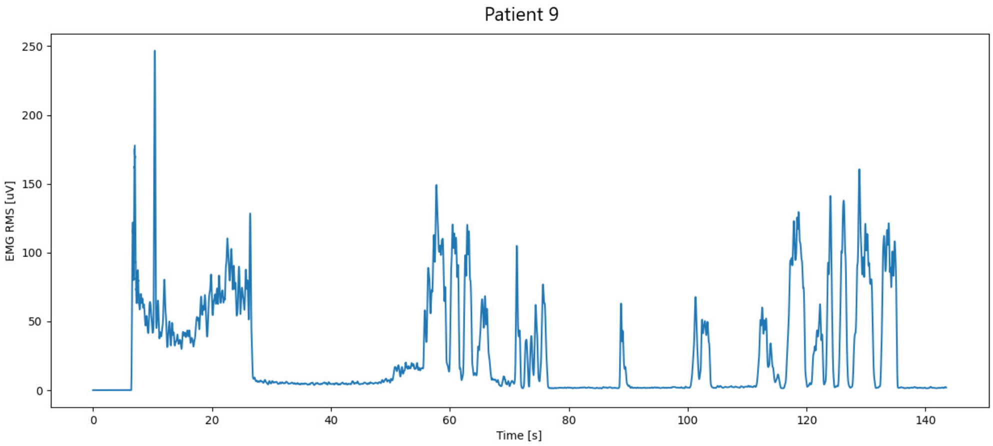
Patient 9
EMG RMS [uV]
250
200
150
100
50
0
0
20
40
60
80
100
120
140
Time [s]

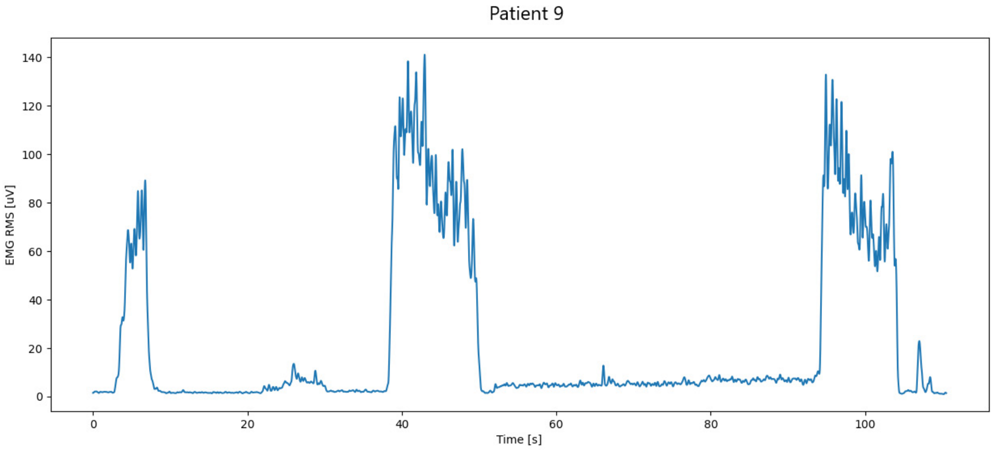
Patient 9
EMG RMS [uV]
140
120
100
80
60
40
20
0
0
20
40
60
80
100
Time [s]

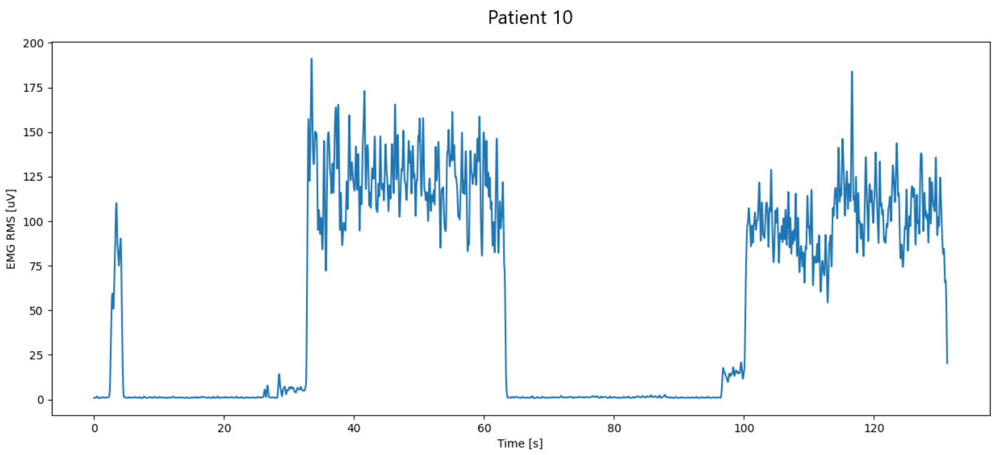
Patient 10
EMG RMS [uV]
Time [s]
0
25
50
75
100
125
150
175
200
0
20
40
60
80
100
120

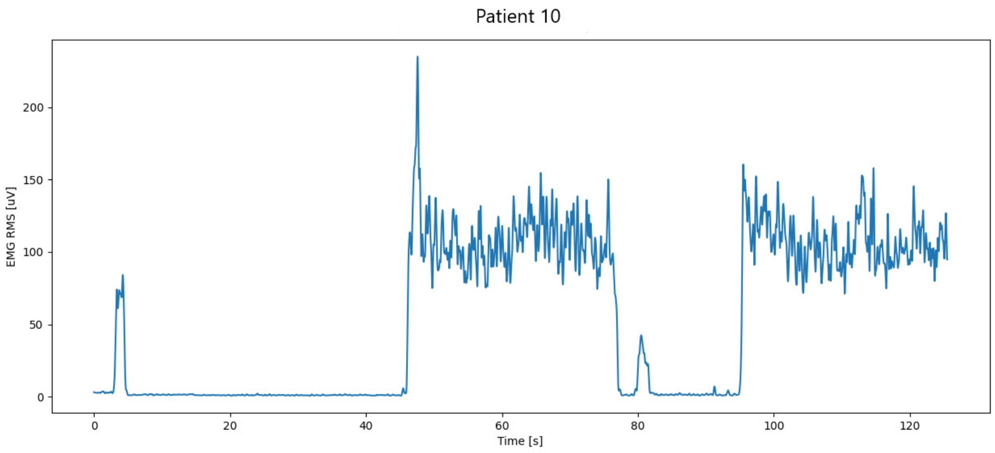
Patient 10
EMG RMS [uV]
Time [s]
0
50
100
150
200
0
20
40
60
80
100
120

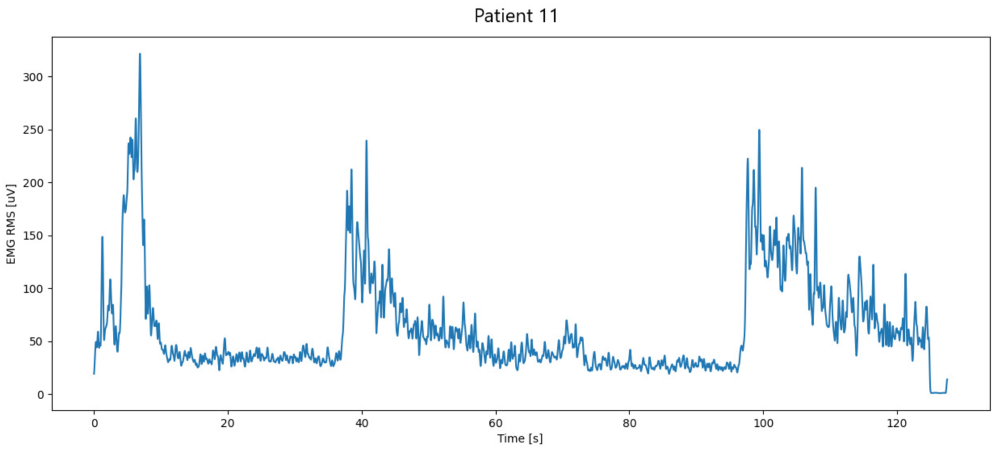
Patient 11
EMG RMS [uV]
Time [s]
0
50
100
150
200
250
300
0
20
40
60
80
100
120

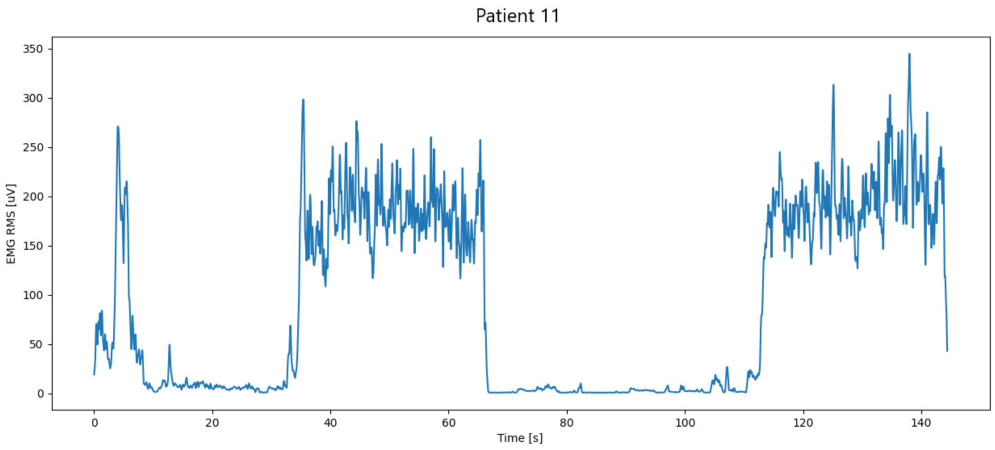
Patient 11
EMG RMS [uV]
Time [s]
0
50
100
150
200
250
300
350
0
20
40
60
80
100
120
140

Patient 12

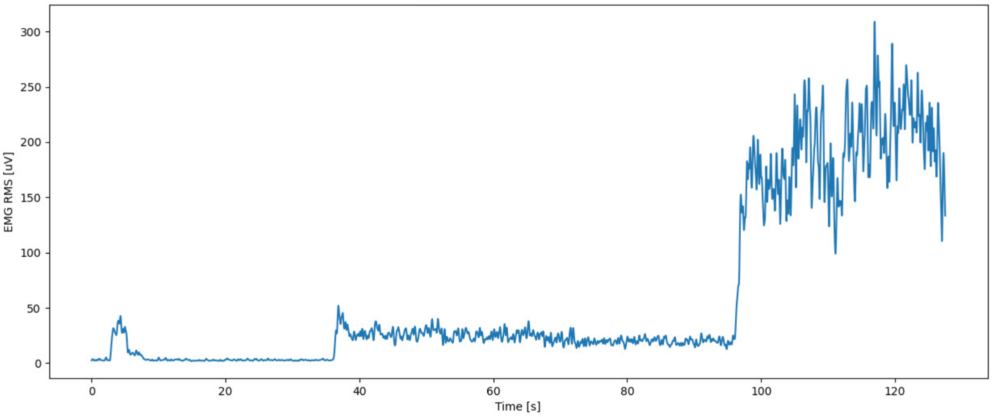

Patient 12

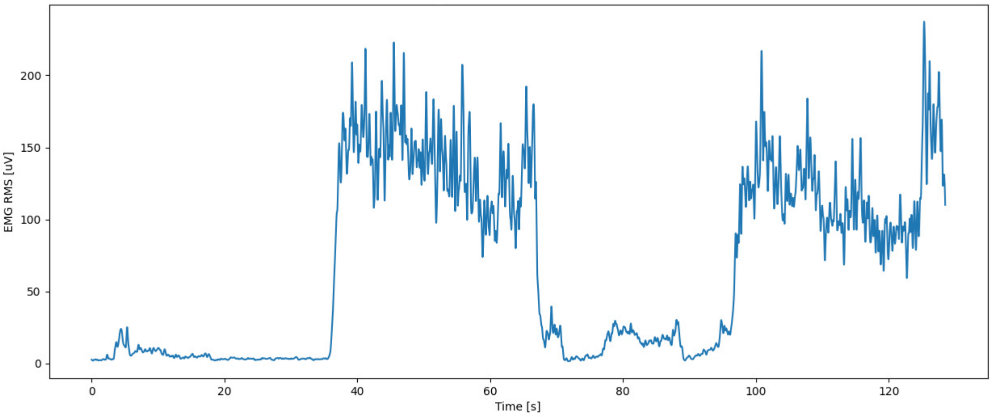

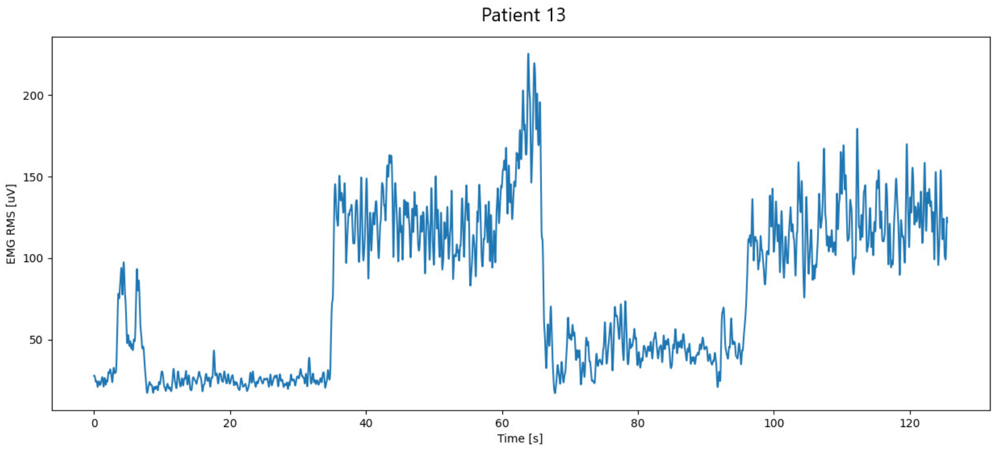

Patient 13
EMG RMS [uV]
Time [s]

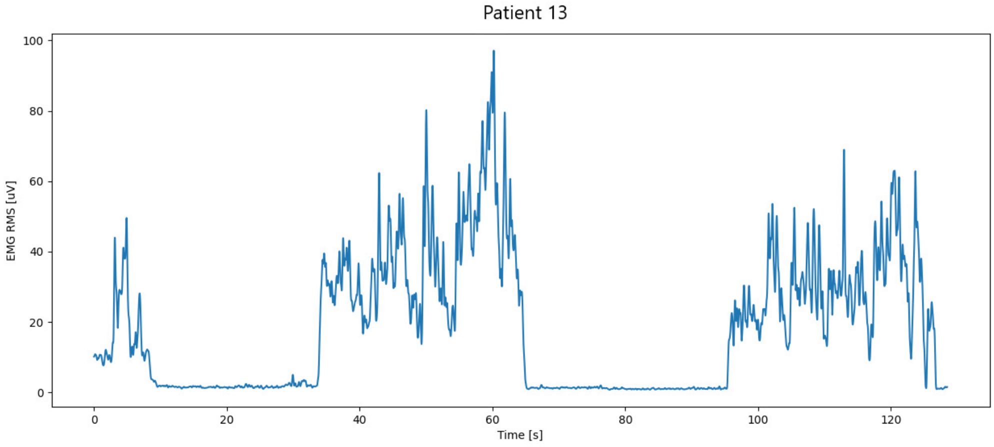

Patient 13
EMG RMS [uV]
Time [s]

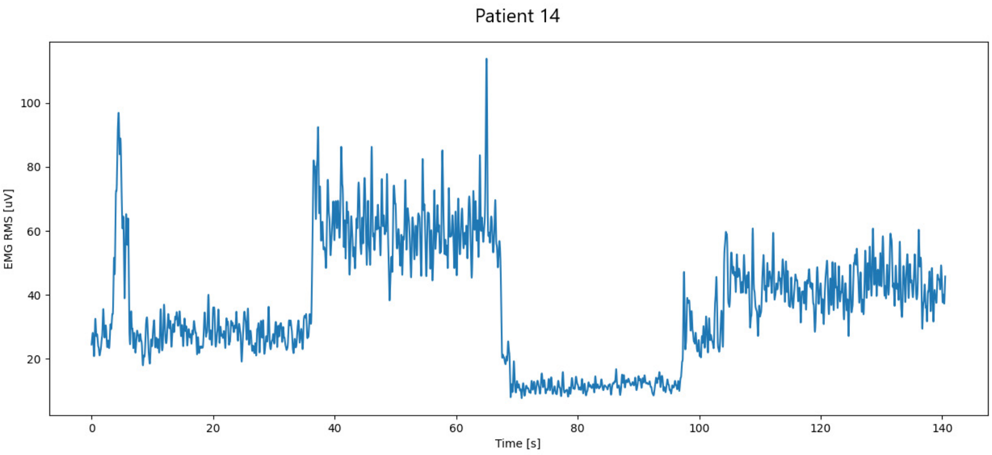

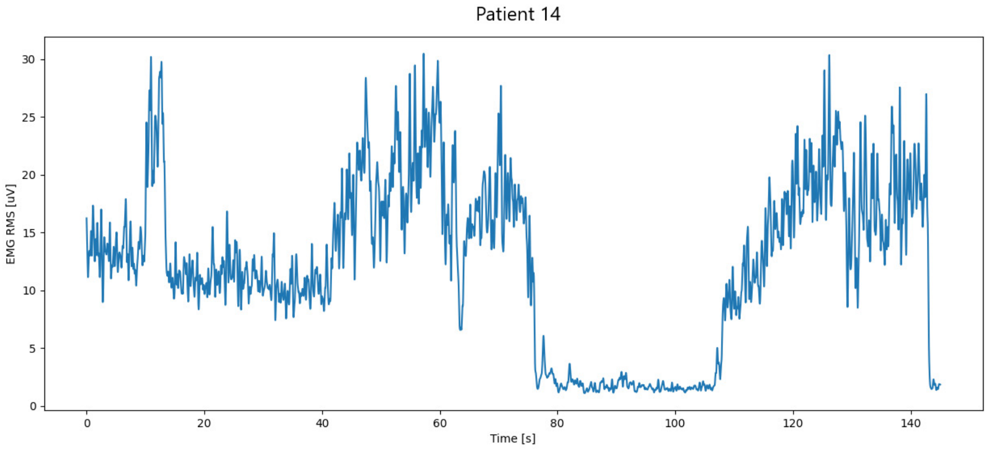

Control Group
